# Supplementary material for: Complexome profiling of the Chlamydomonas psb28 mutant reveals TEF5 as an early PSII assembly factor
Source: Plant Cell. 2025 Mar 17;37(6):koaf055. doi: 10.1093/plcell/koaf055 (PMC12164590; doi:10.1093/plcell/koaf055)
Supplement: koaf055_Supplementary_Data [file koaf055_supplementary_data.zip › psb28_interactive.zip!/plots/intensity/qualityPlots/peptides/ATP4_Cre11.g467707.t1.1.html]

### Cre11.g467707.t1.1

Trivial name: ATP4  
SeqCoverage [median]: 35.10  
Mass: 21.17 kDa  
  
 

| Sequence | MissedCleavage | Mass [Da] | wtIntensity | muIntensity | Score |
| --- | --- | --- | --- | --- | --- |
| ANHVPVIAQLRPGIVELSSGADTEK | 1 | 2600.39 | 5747.54 | 15725.98 | 60.99 |
| DGVTLPGIDGYFGVK | 0 | 1536.78 | 13480.06 | 9546.27 | 99.50 |
| EFTEAWNK | 0 | 1023.47 | 2831.19 | 8317.28 | 138.33 |
| KDGVTLPGIDGYFGVK | 1 | 1664.88 | 951.87 | 3769.40 | 106.60 |
| SALAAANSAQGQGDEYDQAANR | 0 | 2206.98 | 48846.69 | 104945.12 | 296.01 |
|  |  |  | 71857.3 | 142304.0 |  |
